# Symptoms of Fern Distortion Syndrome Resulting from Inoculation with Opportunistic Endophytic Fluorescent *Pseudomonas* spp.

**DOI:** 10.1371/journal.pone.0058531

**Published:** 2013-03-13

**Authors:** Joseph W. Kloepper, John A. McInroy, Ke Liu, Chia-Hui Hu

**Affiliations:** Department of Entomology and Plant Pathology, Auburn University, Auburn, Alabama, United States of America; Wageningen University and Research Centre, The Netherlands

## Abstract

**Background:**

Fern Distortion Syndrome (FDS) is a serious disease of Leatherleaf fern (*Rumohra adiantiformis*). The main symptom of FDS is distortion of fronds, making them unmarketable. Additional symptoms include stunting, irregular sporulation, decreased rhizome diameter, and internal discoloration of rhizomes. We previously reported an association of symptoms with increased endophytic rhizome populations of fluorescent pseudomonads (FPs). The aim of the current study was to determine if FPs from ferns in Costa Rica with typical FDS symptoms would recreate symptoms of FDS.

**Methodology and Findings:**

Greenhouse tests were conducted over a 29-month period. Micro-propagated ferns derived from tissue culture were first grown one year to produce rhizomes. Then, using an 8×9 randomized complete block experimental design, 8 replicate rhizomes were inoculated by dipping into 9 different treatments before planting. Treatments included water without bacteria (control), and four different groups of FPs, each at a two concentrations. The four groups of FPs included one group from healthy ferns without symptoms (another control treatment), two groups isolated from inside rhizomes of symptomatic ferns, and one group isolated from inside roots of symptomatic ferns. Symptoms were assessed 12 and 17 months later, and populations of FPs inside newly formed rhizomes were determined after 17 months. Results showed that inoculation with mixtures of FPs from ferns with FDS symptoms, but not from healthy ferns, recreated the primary symptom of frond deformities and also the secondary symptoms of irregular sporulation, decreased rhizome diameter, and internal discoloration of rhizomes.

**Conclusions:**

These results suggest a model of causation of FDS in which symptoms result from latent infections by multiple species of opportunistic endophytic bacteria containing virulence genes that are expressed when populations inside the plant reach a minimum level.

## Introduction

Leatherleaf fern (*Rumohra adiantiformis*) is a valuable ornamental plant used in cut flower arrangements because of its long shelf life and symmetrical pyramidal shaped fronds. Areas with substantial production of Leatherleaf fern include Florida, Costa Rica, and other countries in Central America. Since the 1980s, distortions of ferns and other symptoms of damage were reported by growers. The disease was termed fern distortion syndrome (FDS) in 2010 [1]. The main aboveground symptoms of FDS are twisting and distortions of fronds which make fronds unmarketable. Other FDS symptoms include an irregular sporulation pattern and decreased rhizome diameter with occasional internal discoloration [1].

The incidence and severity of FDS was associated with significantly greater populations of fluorescent pseudomonads inside rhizomes of plants with distorted fronds than plants with normal-appearing fronds [1]. From the results, a model was proposed [1] whereby FDS results from latent infections by deleterious fluorescent pseudomonads that induce damage when a threshold population is reached. This model would explain reports from growers that FDS is spread by vegetative propagation of rhizomes from diseased fields.

However, two main questions arise from the model. First, what is the trigger or inducing factor for increase in populations of endophytic deleterious pseudomonads? Fern growers noted that increased distortions of fronds in Florida were reported to coincide with the widespread use of Benlate systemic fungicide [2]. Support for the suggestion that Benlate could be an inducing factor for FDS came from a recent study [3] showing that 24 months after treatment with Benlate, distortions of frond growth and other symptoms associated with FDS were present along with increased endophytic populations of fluorescent pseudomonads inside rhizomes. The second question arising from the model is do fluorescent pseudomonads isolated from ferns with symptoms of FDS recreate symptoms upon inoculation into asymptomatic ferns?

Accordingly, the main objective of the current study was to determine if fluorescent pseudomonads isolated from plants with typical symptoms of FDS in Costa Rica recreate the primary distortion symptoms upon inoculation into asymptomatic ferns. In this study, the rhizomes used for inoculation with test bacteria were obtained from the field in Florida. For the current study, rhizomes were collected from micro-propagated ferns so that all inoculations could be done on fern plants of identical age to increase precision of results and to eliminate the potential for indigenous bacteria in an established fernery to contribute to the symptoms that develop following inoculation.

## Materials and Methods

### Sources of ferns and growth conditions

Leatherleaf fern plants initiated from tissue culture of growing tips of healthy rhizomes were obtained from Casa Flora, Dallas, Texas. Plants were maintained as per the methods of Strandberg [4] using large plastic pots and a planting mix consisting of 60% pine bark, 20% peat, and 20% sand amended with dolomitic lime and micro-nutrients. Ferns were grown in the greenhouse under a shade fabric that provided 55% shade, and they were fertilized weekly with 250 ppm of water soluble 20-10-20 fertilizer. Micronutrients were applied every 4 months. Ferns from tissue culture were grown for 12 months to allow formation of rhizomes. After inoculation of rhizomes (described below), plants were maintained in the same manner in the greenhouse.

### Isolation, identification, and characterization of fluorescent pseudomonads isolated from field-grown ferns and used in inoculations

#### A. Isolation

The fluorescent pseudomonads used to inoculate rhizomes (Table 1) were isolated either from ferns with FDS symptoms in Costa Rica or from healthy-appearing ferns without FDS symptoms in Florida. As listed in Tables 1 and 2, the bacteria were isolated either from inside rhizomes (endophytic bacteria) or from the rhizosphere. Methods of isolation were previously described in detail [1]. In summary, endophytic fluorescent pseudomonads (treatments 2A, 2B, 3A, 3B, 4A, and 4B in Tables 1 and 2) were isolated by removing a section 4 cm in length from the growing end of each rhizome. These sections were soaked for 3 min in 70% ethanol followed by flaming. After cooling, rhizomes were pressed onto agar plates of 10% tryptic soy agar to confirm that surface bacteria were eliminated. Sections of surface disinfested rhizomes weighing 1.5–2.5 g were aseptically triturated for 1 min in 10 ml of sterile distilled water in autoclaved stainless steel ball-mill containers using a Kleco tissue pulverizer (Garcia Manufacturing, Visalia, California). Serial dilutions to 10^−2^ were made and plated onto 50% King's medium B (KB). Rhizosphere bacteria (treatments 5A and 5B in Tables 1 and 2) were isolated by shaking field-collected rhizomes with attached roots to remove loosely attached soil. Then 2.0 g of roots with remaining attached soil were shaken for 20 min in 50 ml sterile water in 125 ml Erlenmeyer flasks. Serial dilutions were made and plated onto KB. After incubation for 48 hours at 28°C, representative colony types that fluoresced under ultraviolet light were selected, purified, and stored at −80°C.

**Table 1:** Identification and characterization of fluorescent pseudomonads used to inoculate fern rhizomes to determine development of symptoms of FDS.

Treatment number in inoculation test	Strain number	Origin^1^	Closest matching type strains^2^	Phylogenetic cluster^3^	HR in tobacco^4^	Rot of potato slice^5^	IAA production (µg/ml)^6^
2A and B	B1	**HRZ-1**	*Pseudomonas umsongensis* (0.963)*Pseudomonas jessenii* (0.958)	C	−	+	6.53
2A and B	B2	**HRZ-2**	*Pseudomonas moraviensis* (0.969)*Pseudomonas koreensis* (0.964)	B	−	+	11.67
2A and B	B4	**HRZ-3**	*Pseudomonas vancouverensis* (0.962)*Pseudomonas umsongensis* (0.951)	C	−	+	26.37
2A and B	B8	**HRZ-4**	*Pseudomonas moraviensis* (0.969)*Pseudomonas koreensis* (0.964)	A	−	+	10.46
2A and B	B9	**HRZ-5**	*Pseudomonas fulva/putida* (0.965)*Pseudomonas taiwanensis* (0.962)	D	−	−	21.27
2A and B	B11	**HRZ-6**	*Pseudomonas jessenii* (0.962)*Pseudomonas vancouverensis* (0.950)	C	−	−	17.66
2A and B	B12	HRZ-7	*Pseudomonas vancouverensis* (0.962)*Pseudomonas umsongensis* (0.951)	C	−	−	21.71
2A and B	B13	**HRZ-8**	*Pseudomonas umsongensis* (0.972)*Pseudomonas jessenii* (0.964)	C	−	−	24.64
2A and B	B20	**HRZ-9**	*Pseudomonas taiwanensis* (0.986)*Pseudomonas monteilii* (0.985)	D	−	−	21.47
2A and B	B21	**HRZ-10**	*Pseudomonas jessenii* (0.965)*Pseudomonas vancouverensis* (0.963)	C	−	−	17.85
2A and B	B24	**HRZ-11**	*Pseudomonas vancouverensis* (0.974)*Pseudomonas umsongensis* (0.966)	C	−	+	20.76
2A and B	B27	**HRZ-12**	*Pseudomonas vancouverensis* (0.964)*Pseudomonas umsongensis* (0.950)	C	−	−	18.62
2A and B	B35	**HRZ-13**	*Pseudomonas vancouverensis* (0.977)*Pseudomonas umsongensis* (0.972)	C	−	−	18.82
2A and B	B38	HRZ-14	*Pseudomonas moraviensis* (0.969)*Pseudomonas koreensis* (0.964)	B	−	−	11.77
2A and B	B41	**HRZ-15**	*Pseudomonas jessenii* (0.966)	C	−	+	20.41
2A and B	B42	**HRZ-16**	*Pseudomonas moraviensis* (0.969)*Pseudomonas koreensis* (0.964)	B	−	−	8.45
2A and B	B45	**HRZ-17**	*Pseudomonas moraviensis* (0.969)*Pseudomonas koreensis* (0.964)	B	−	−	7.87
3A and B	CRI5	**SRZ-1**	*Pseudomonas moraviensis* (0.977)*Pseudomonas koreensis* (0.974)	B	+	++	11.14
3A and B	CRI6	**SRZ-2**	*Pseudomonas taiwanensis* (0.967)*Pseudomonas monteilii* (0.965)	D	−	−	23.06
3A and B	CRI7	**SRZ-3**	*Pseudomonas vancouverensis* (0.961)*Pseudomonas moraviensis* (0.959)	A	++	+	13.19
3A and B	CRI8	**SRZ-4**	*Pseudomonas chlororaphis* (0.974)*Pseudomonas moraviensis* (0.960)	B	++	++	12.35
3A and B	CRI9	**SRZ-5**	*Pseudomonas umsongensis* (0.953)*Pseudomonas moraviensis* (0.951)	B	++	+	12.28
3A and B	CRI10	**SRZ-6**	*Pseudomonas vancouverensis* (0.964)*Pseudomonas moraviensis* (0.955)	A	++	++	9.76
3A and B	CRI12B	**SRZ-7**	*Pseudomonas vancouverensis* (0.951)*Pseudomonas umsongensis* (0.949)	B	++	++	12.05
3A and B	CRI26	SRZ-8	*Pseudomonas umsongensis* (0.953)*Pseudomonas moraviensis* (0.951)	B	+	++	8.26
3A and B	CRI27	SRZ-9	*Pseudomonas umsongensis* (0.953)*Pseudomonas moraviensis* (0.951)	B	+	+	11.56
3A and B	CRI28	SRZ-10	*Pseudomonas umsongensis* (0.953)*Pseudomonas moraviensis* (0.951)	B	+	+	10.66
3A and B	CRI29	SRZ-11	*Pseudomonas vancouverensis* (0.961)*Pseudomonas moraviensis* (0.959)	A	+	++	7.71
4A and B	CRI13	SRZ-12	*Pseudomonas umsongensis* (0.953)*Pseudomonas moraviensis* (0.951)	B	+	++	10.27
4A and B	CRI14	SRZ-13	*Pseudomonas vancouverensis* (0.961)*Pseudomonas moraviensis* (0.959)	A	+	−	7.78
4A and B	CRI15	SRZ-14	*Pseudomonas umsongensis* (0.953)*Pseudomonas moraviensis* (0.951)	B	−	++	7.43
4A and B	CRI16	SRZ-15	*Pseudomonas vancouverensis* (0.961)*Pseudomonas moraviensis* (0.959)	A	−	++	8.14
4A and B	CRI17	SRZ-16	*Pseudomonas vancouverensis* (0.961)*Pseudomonas moraviensis* (0.959)	A	−	++	9.05
5A and B	CRI62	SRS-1	*Pseudomonas vancouverensis* (0.951)*Pseudomonas umsongensis* (0.949)	B	+	+	7.68
5A and B	CRI63	SRS-2	*Pseudomonas umsongensis* (0.953)*Pseudomonas moraviensis* (0.951)	B	+	++	9.91
5A and B	CRI64	SRS-3	*Pseudomonas vancouverensis* (0.961)*Pseudomonas moraviensis* (0.959)	A	+	++	6.02
5A and B	CRI65	SRS-4	*Pseudomonas chlororaphis* (0.974)*Pseudomonas moraviensis* (0.960)	B	+	++	8.39
5A and B	CRI66c	SRS-5	*Pseudomonas vancouverensis* (0.961)*Pseudomonas moraviensis* (0.959)	A	+	++	6.46
5A and B	CRI67	SRS-6	*Pseudomonas umsongensis* (0.953)*Pseudomonas moraviensis* (0.951)	B	+	−	11.50
5A and B	CRI68	SRS-7	*Pseudomonas vancouverensis* (0.951)*Pseudomonas umsongensis* (0.949)	B	−	+	10.80
5A and B	CRI69	SRS-8	*Pseudomonas vancouverensis* (0.964)*Pseudomonas moraviensis* (0.955)	A	−	+	8.00
5A and B	CRI70	SRS-9	*Pseudomonas chlororaphis* (0.974)*Pseudomonas moraviensis* (0.960)	B	−	+	9.66
5A and B	CRI71	SRS-10	*Pseudomonas vancouverensis* (0.961)*Pseudomonas moraviensis* (0.959)	A	+	+	9.10
5A and B	CRI72b	**SRS-11**	*Pseudomonas citronellolis* (0.981)	E	+	+	7.79
5A and B	CRI73	SRS-12	*Pseudomonas vancouverensis* (0.961)*Pseudomonas moraviensis* (0.959)	A	+	+	7.40
5A and B	CRI74	SRS-13	*Pseudomonas fulva/putida* (0.965)*Pseudomonas taiwanensis* (0.962)	D	+	−	16.67
5A and B	CRI75a	SRS-14	*Pseudomonas citronellolis* (0.981)	E	+	−	7.79

1 HRZ = Inside rhizomes of healthy-appearing ferns from a fernery in Florida without history of Benlate use; SRS = Rhizosphere (roots and rhizomes) of symptomatic ferns in Costa Rica; SRZ = inside rhizomes of symptomatic ferns in Costa Rica. Bold indicates isolates that have unique 16S rRNA gene sequences.

2 Identified using phylogenetic analysis of 16S rRNA gene sequencing. Numbers in parentheses indicate the “seqmatch score”. These are the number of unique 7-base oligomers shared between each strain sequence and the indicated RDP sequence divided by the lowest number of unique oligos in either of the two sequences.

**Figure 1:**
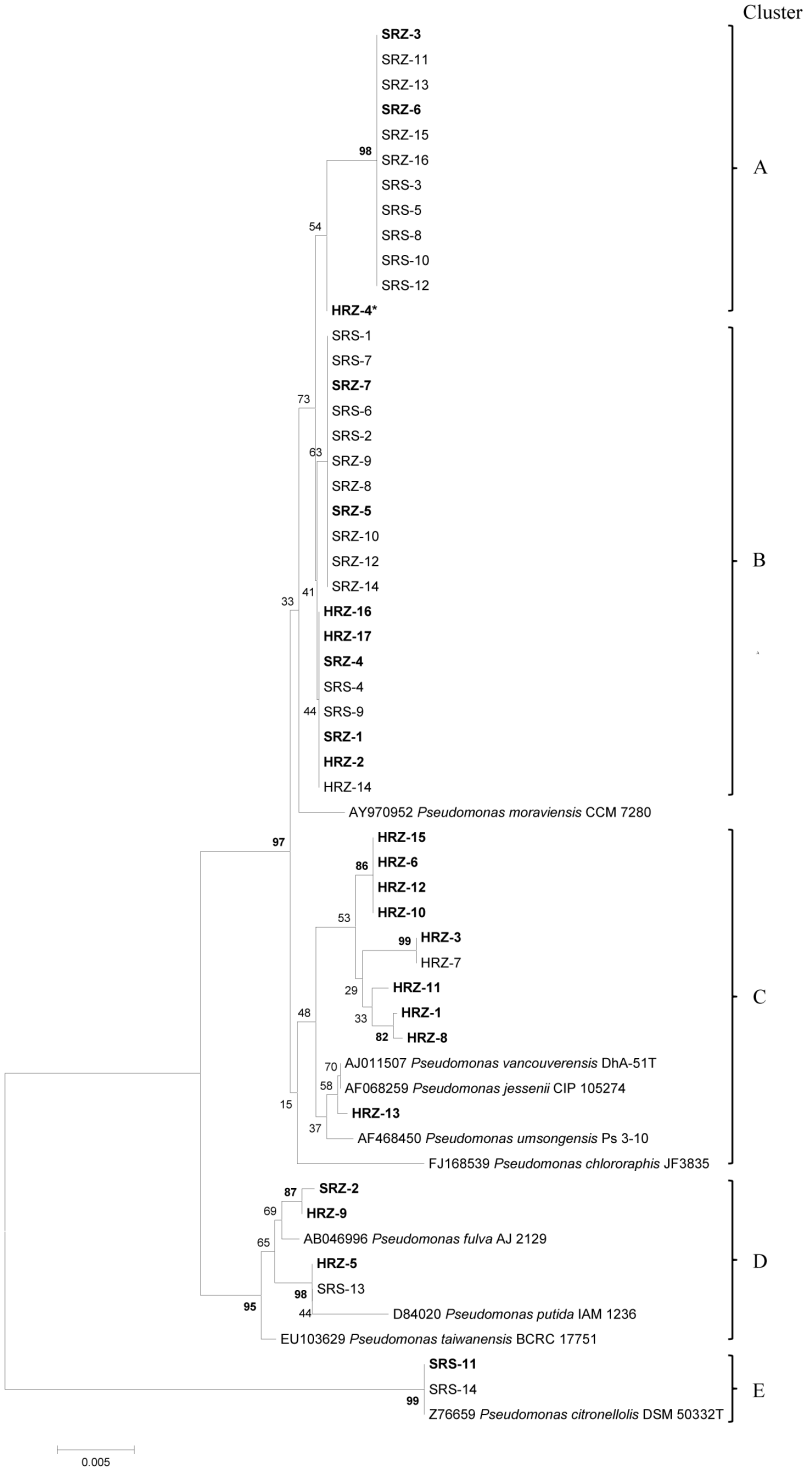
Neighbor-joining (NJ) bootstrap consensus tree of partial 16S rRNA gene (1412 bp). This Figure illustrates the phylogenetic relationship among fluorescent *Pseudomonas* spp. used to inoculate fern rhizomes. Strain codes are listed in legend of Table 1. The analyses were done with MEGA version 5 [6], and the numbers at each node represent bootstrap value (1000 replicates). Scale bar: substitutions/site.

3 The phylogenetic clusters are shown in Figure 1.

4 Tobacco hypersensitive test; ++ indicates dry necrosis in 24–36 hr after inoculation; + indicates wet necrosis in 48 hr.

5 Measurement of pectinolytic activity, where ++ indicates soft rot of potato slice 24 hr after inoculation; + indicates soft rot 48 hr after inoculation.

6 Production of IAA was quantified as described in the methods.

**Table 2:** Summary of the inoculated fluorescent pseudomonads in each treatment group belonging to each phylogenetic cluster shown in Figure 1.

	Treatment					
Phylogenetic Clusters	HRZ (treatment 2)	SRZ (treatment 3)	SRZ (treatment 4)	SRS (treatment 5)	No. of Isolates	Closest matches of type strain(s) (with highest SeqMatch score)^1^
**A**	1	3	3	5	12	*Pseudomonas vancouverensis* (0.964); *Pseudomonas moraviensis* (0.969)
**B**	4	7	2	6	19	*Pseudomonas moraviensis* (0.977); *Pseudomonas chlororaphis* (0.974); *Pseudomonas umsongensis* (0.953); *Pseudomonas vancouverensis* (0.951)
**C**	10	0	0	0	10	*Pseudomonas vancouverensis* (0.977); *Pseudomonas umsongensis* (0.972); *Pseudomonas jessenii* (0.966)
**D**	2	1	0	1	4	*Pseudomonas taiwanensis* (0.986); *Pseudomonas fulva/putida* (0.965)
**E**	0	0	0	2	2	*Pseudomonas citronellolis* (0.981)

1 Taxa of fluorescent pseudomonads in the Ribosomal Database Project II with 16S rRNA gene sequences most similar to those isolates in each cluster.

#### B. Identification

Taxonomic classification of each strain of the fluorescent pseudomonads used to inoculate rhizomes was based on the partial sequence of 16S rRNA gene. DNA was extracted and amplified with universal bacterial primers: 8F (5′- AGAGTTTGATCCTGGCTCAG -3′) and 1492R (5′- ACGGCTACCTTGTTACGACTT- 3′). PCR was performed using Lucigen EconoTaq Plus Green 2× master mix (Lucigen Corp.) with the following cycling parameters: initial denaturation at 95°C for 5 min; 31 cycles of 94°C for 1 min, 57°C for 45 sec, 70°C for 2 min; and a final extension at 70°C for 10 min.

DNA sequences were blasted against the type strains in the ribosomal database project using SEQMATCH to identify bacterial taxa of each strain. The partial 16S rRNA gene sequences of the inoculated strains and closely related type strains of *Pseudomonas* spp. were aligned with ClustalW and phylogenetic analysis was conducted with MEGA version 5 [5]. Data were analyzed by neighbor-joining (NJ) and the distance matrix was calculated by the Kimura 2-K parameter model. A bootstrap test with 1,000 replicates was used to estimate the confidence of the phylogenetic tree. After constructing the phylogenetic tree, a summary table was prepared to show the percentage of isolates from the three sample sources (rhizomes from healthy appearing ferns [HRZ], rhizomes from symptomatic ferns [SRZ], and rhizosphere of symptomatic ferns [SRS]).

#### C. Characterization, elicitation of HR, potato slice maceration, and IAA production

All of the strains of fluorescent pseudomonads used to inoculate rhizomes were evaluated for three traits previously associated with bacterial potential to damage plants. The classical tobacco hypersensitive test system (HR), which has been used to determine the phytopathogenic potential of plant-associated bacteria [6], was adapted from Campbell et al. [7] and Gardner et al. [8]. Tobacco cv. Samsun was used, and the test was conducted using the second and third fully expanded leaves on various plants. Suspensions of bacterial strains in sterile distilled water at 10^8^ to 10^9^ cfu/ml were injected into the interveinal area of tobacco leaves. A sterile water control was included in each test. Results were recorded at 24 and 48 hours after inoculation [8]. The presence of dry necrosis in the injected area at 24 hours was recorded as a ++ reaction, and the presence of wet necrosis at 48 hours after inoculation was recorded as a + reaction.

The potato slice maceration test was used as a measure of pectinolytic enzyme activity. The procedure was modified from González et al. [9]. Bacterial strains were streaked onto the top of potato slices placed in petri dishes with 5 ml sterile water around the bottom of the slices. One non-inoculated potato slice was included from each tuber used to produce the slices. Soft rot development was examined after incubation at room temperature for 24 and 48 hours.

Bacterial production of indole-3 acetic acid (IAA) was determined qualitatively and quantitatively based upon previously described methods [10], [11]. Test bacterial strains were grown for 24 hours in nutrient broth with shaking at room temperature. For qualitative production, 10 µl of bacterial culture was inoculated into 1.0 ml of minimal salt medium amended with 5 mM/L of L-tryptophan and then incubated with shaking for 48 hours. The culture was centrifuged at 12,000 rpm for 5 min, and then 50 µl of the supernatant was added to 100 µl of FeCl_3_-HClO_4_ reagent, which was kept in the dark. Development of a pink color in 25 min indicated production of IAA. Production of IAA was quantified by inoculating 200 µl of a 24 hour nutrient broth culture into 20 ml of minimal salt medium amended with 5 mM/L of L-tryptophan and then shaking at room temperature for 48 hours. After centrifuging at 12,000 rpm for 5 min, 1.0 ml of the supernatant was added to 2 ml FeCl_3_-HClO_4_ reagent. After 25 min, absorbance at 530 nm was determined in a uv-spectrophotometer, and the concentration of IAA was determined in µg/ml using a standard curve.

### Inoculation of rhizomes and effects of fluorescent pseudomonads on development of symptoms of FDS

An experiment was designed to determine if fluorescent pseudomonads isolated from plants with typical symptoms of FDS in Costa Rica recreate the primary distortion symptoms upon inoculation into asymptomatic ferns. The experiment was a randomized complete block design with 9 treatments and 8 replications per treatment, for a total experimental number of 72 plants. Treatments, shown in Table 1, included a water-inoculated control and four groups of fluorescent pseudomonads (treatments 2–5) each at two bacterial concentrations (log 6.0 and log 8.0 cfu/ml). The strains of fluorescent pseudomonads in treatment 2 were isolated from inside rhizomes of healthy-appearing, asymptomatic ferns collected in a fernery in Florida with no history of using Benlate fungicide. Strains in treatments 3 and 4 were isolated from inside rhizomes of ferns expressing symptoms of FDS in Costa Rica. Strains in treatment 5 were from the rhizosphere of ferns expressing symptoms of FDS in Costa Rica. Bacterial concentrations of each treatment group were prepared by growing bacterial strains individually on KB for 48 hours at 28°C and then adjusting optical density with sterile water to obtain the stated cell densities.

Rhizomes of uniform size from ferns derived from tissue culture and grown 12 months in the greenhouse at Auburn University, as described above, were selected, with 8 rhizomes per treatment. Rhizomes were dipped into treatments for 5 minutes immediately prior to planting in the greenhouse. Pots were randomized in the greenhouse and were grown as described above.

The effect of bacterial treatments on the development of symptoms of FDS was assessed at 12 months after inoculation in a non-destructive sampling of the fern plants. The mean FDS severity was determined for the youngest fully developed frond on each plant by using the four point scale developed previously [1] for assessing the severity of distortions in commercial ferneries. With this scale, 0 indicates normal appearance with symmetrical frond; 1 indicates slight distortion of frond shape with bending of the frond tip or some twisting of the frond rachis; 2 indicates more severe distortion of frond shape with clear asymmetrical shape overall and specifically a distorted appearance of the frond tip; and 3 indicates severe twisting of rachis and loss of triangular shape of frond. Additional measurements made to quantify aspects of fern growth related to symptoms of FDS included assessing the mean height of plants, mean width of the largest frond on each plant, mean number of the 5 largest fronds with a twisted rachis, mean number of the 5 largest fronds with a pyramidal shape, number of plants with dwarfing, mean number of the 5 largest fronds with leaflets twisted out of the plane of the frond, and the number of plants with crispy, thickened fronds.

At 17 months after inoculation, the effects of bacterial treatments on plant growth and the population densities of fluorescent pseudomonads inside rhizomes (described below) were determined by destructively sampling the inoculated plants. Measurement of plant growth included total plant fresh weight, number of rhizomes per plant, the caliper of three rhizomes per plant (as a way to assess rhizome size), and the dry weights of roots and fronds.

For each measurement conducted at 12 and 17 months after planting, data were analyzed for significant treatment effects using two-way analysis of variance with SAS. When a significant F value was determined, LSD values were calculated at *P* = 0.01.

### Reisolation of fluorescent pseudomonads 17 months after inoculation of healthy rhizomes: quantification of endophytic populations inside rhizomes, identification, and characterization

An isolation of bacteria was conducted at 17 months after inoculation to quantify and characterize fluorescent pseudomonads inside rhizomes of plants that developed symptoms of FDS (treatments 3A, 3B, 4A, 4B, and 5B). From these treatments, three rhizomes from each replicate plant of each treatment were selected for sampling. After surface disinfestation as described above, a combined sample of 0.5–1.5 g of rhizome tissue was made for each replication. Samples were processed as described above, and serial 10-fold dilutions were prepared and plated on 50% KB. After incubation for 48 hours at 28°C, fluorescent colonies were counted under an ultraviolet light, and the mean log cfu/g of rhizome was calculated for each treatment. Data were analyzed for significant treatment effects using two-way analysis of variance as described above.

A collection was made of 350 strains of fluorescent pseudomonads from symptomatic ferns. Combined collections of 100 strains from treatments 3A and 3B, 100 from treatment 4A and 4B, and 100 from treatments 5A and 5B were made from the dilution plates used to quantify the fluorescent pseudomonads inside rhizomes. In addition, a collection of 50 strains (labeled in results as 5INT) was made from inside rhizomes from treatments 5A and 5B that showed internal discoloration, which was previously reported inside rhizomes of ferns expressing symptoms of FDS in Costa Rica (Figure B in Kloepper et al. [1]). Each of the 350 strains was identified using phylogenetic analysis of 16S rRNA gene sequencing, as described above. All strains were also tested for elicitation of HR, pectinolytic activity (maceration of potato slice), and production of IAA as described above.

## Results

### Isolation, identification, and characterization of fluorescent pseudomonads originally isolated from field-grown ferns and used in inoculations

#### A. Identification

The identification of the strains of fluorescent pseudomonads isolated from field-grown ferns with or without symptoms of FDS (Table 1, Figure 1, and Table 2) revealed clear differences between strains from healthy ferns and strains from symptomatic ferns. For example, 88% of the strains from healthy rhizomes (treatment 2) had unique 16S rRNA gene sequences, compared to 47% of strains from symptomatic rhizomes (treatments 3+4) and 7% of strains from the rhizosphere of symptomatic ferns (Table 2). The phylogenetic analysis revealed that the strains from all sources fit into five phylogenetic clusters (Figure 1). Differences were noted in the frequency with which strains of pseudomonads from different treatments belonged to these clusters. Of the strains isolated from rhizomes of asymptomatic ferns, 58.8% belonged to cluster C. However, none of the strains from rhizomes or the rhizosphere of symptomatic ferns belonged to this cluster. Strains that fit into clusters A and B were dominant among strains from symptomatic ferns, representing 90.9% of strains from treatment 3, 100% from treatment 4, and 78.6% from treatment 5. In contrast, 41.7% of strains from asymptomatic fern rhizomes fit into these same clusters.

#### B. Characterization, elicitation of HR, potato slice maceration, and IAA production

Results of the characterization of the fluorescent strains isolated from the field-grown ferns and used to inoculate rhizomes in the greenhouse revealed differences among the groups of bacteria in the frequency of some traits linked to virulence. For example, none of the pseudomonads isolated from inside rhizomes of healthy-appearing, asymptomatic plants without a history of Benlate use elicited HR in tobacco leaves (Table 1). In contrast, HR was elicited by 77% of the fluorescent pseudomonads isolated from inside rhizomes or from the rhizosphere of diseased, symptomatic ferns within 24–48 hours after inoculation (Table 1). Of these strains, 17% produced the classical HR consisting of a dry, brown interveinal necrosis in 24–36 hours, which is an indication that a bacterial strain is pathogenic [6], and 60% produced a wet, black interveinal necrosis in 48 hours. This black, wet necrosis resembled symptoms of bacterial soft rot caused by pectinolytic enzymes and led us to investigate the frequency of pectinolytic enzyme production via the potato slice maceration test.

With the potato slice maceration test, none of the strains of fluorescent pseudomonads from inside rhizomes of asymptomatic plants had strong pectinolytic activity (causing soft rot of potato slices in 24 hours) (Table 1), while 46% of the strains isolated from inside rhizomes or from the rhizosphere of diseased ferns had strong pectinolytic activity. In addition, the percentage of strains from diseased plants that had weaker pectinolytic activity (causing soft rot in 48 hours) was 83% compared with 35% of the strains from healthy plants (Table 1). Production of IAA *in vitro* was noted for all tested strains (Table 1). In contrast to elicitation of HR and pectinolytic activity, the level of IAA production was not less for strains isolated from healthy plants.

### Effects of fluorescent pseudomonads on development of symptoms of FDS

**Figure 2:**
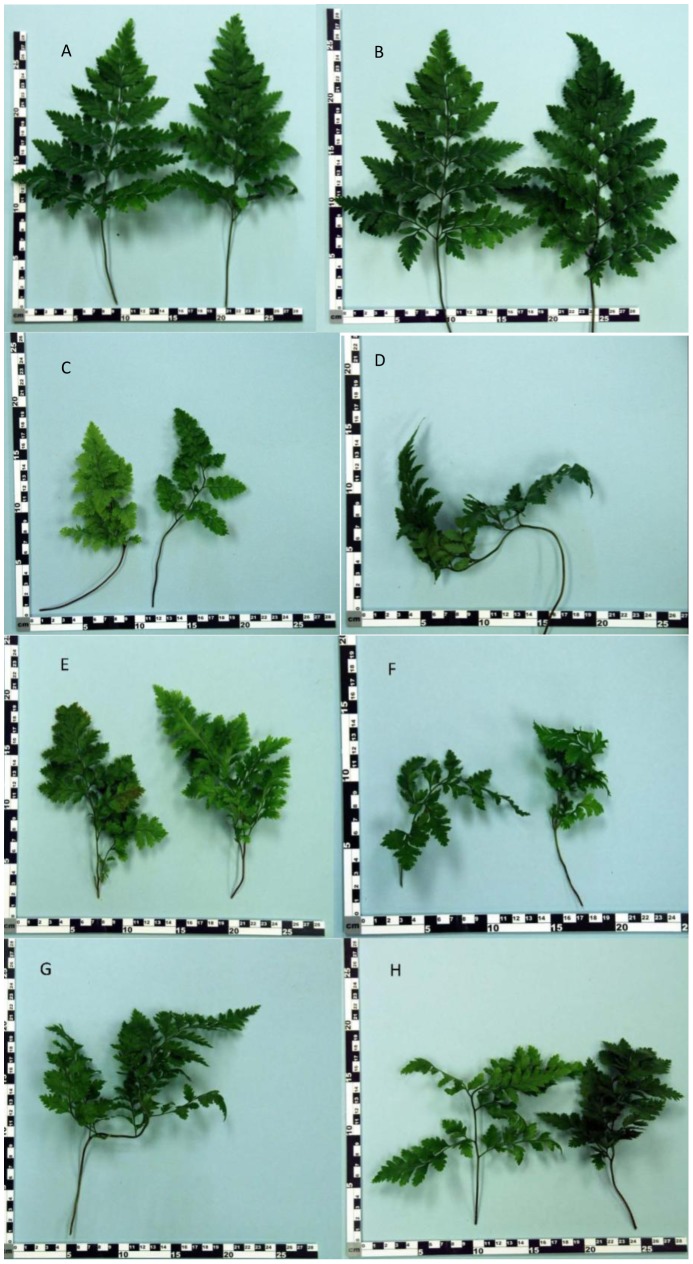
Recreation of FDS symptoms of frond deformities by inoculation with fluorescent pseudomonads from diseased plants. Examples of distortions evident at 12 months after inoculation. A = water control (treatment 1), B = bacteria from inside rhizomes of healthy ferns (treatment 2B), C–F = bacteria from inside rhizomes of ferns with FDS symptoms (treatments 3A, 3B, 4A, and 4B), G–H = rhizosphere bacteria from ferns with FDS symptoms (treatments 5A and 5B).

In commercial ferneries, healthy Leatherleaf ferns produce highly symmetrical growth with a straight to slightly curving central rachis terminating in a definitive tip and having an overall pyramidal shape. The most characteristic reported symptom of FDS is distorted growth of fronds [1] that ranges from the bending of the tip to more severe twisting of the rachis causing loss of the pyramidal shape. In addition, pinnae (the leaf-like segments of a frond) often grow out of the plane of the frond, resulting in a bunching of growth. As affected fronds mature, they can exhibit severely deformed shapes. All of these symptoms were recreated 12 months after inoculation with the strains of fluorescent pseudomonads isolated from diseased but not from healthy plants (Table 3 and Figure 2). Specifically, inoculation with both concentrations of pseudomonads isolated from inside fronds or the rhizosphere of ferns with FDS symptoms (treatments 3A, 3B, 4A, 4B, 5A, and 5B) resulted in statistically significant (*P* = 0.01) increases compared to the controls (treatments 1, 2A, and 2B) in FDS severity, number of largest five fronds with a twisted rachis, and number of largest five fronds with pinnae twisted out of the plane of the frond. All treatments inoculated with bacteria from diseased ferns resulted in a significantly lower number of the largest five fronds with a pyramidal shape, compared to the controls. The corresponding photographs of deformed frond growth resulting from inoculation with pseudomonads from plants with FDS symptoms (Figure 2) show a range of deformities similar to those reported in the field in Costa Rica [1].

**Table 3:** Symptoms of Fern Distortion Syndrome 12 months after inoculation of fern rhizomes with fluorescent pseudomonads isolated from symptomatic and asymptomatic leatherleaf fern.

Treatment number^1^	Source of bacteria and concentration 2	Mean FDS severity rating^3^^,^ 4 ^,^	Mean height (cm) of plants^4^	Mean width (cm) of largest frond per plant^4^	Mean no. of largest 5 fronds with twisted rachis^4^	Mean no. of largest 5 fronds with pyramidal shape^4^	No. of plants with dwarfing	Mean no. of largest 5 fronds with pinnae twisted out of the plane of the frond^4^	No. of plants with crispy, thickened fronds
1	No bacteria	0.38 C	28.1 A	8.5 B	0.25 D	4.63 A	0	0.25 C	0
2A	HRZ log 6.0	0.25C C	28.9 A	9.6 A	0.38 D	4.75 A	0	0.13 C	0
2B	HRZ log 8.0	0.38 C	28.5 A	9.6 A	0.63 D	4.50 A	0	0.25 C	0
3A	SRZ log 6.0	2.38 AB	20.1 B	6.6 C	4.75 A	0.38 C	6	3.25 AB	2
3B	SRZ log 8.0	2.63 A	16.5 B	5.0 D	4.75 A	0.13 C	6	4.00 A	3
4A	SRZ log 6.0	2.25 AB	21.3 B	5.5 D	4.00 AB	0.63 C	4	3.38 AB	2
4B	SRZ log 8.0	2.38 AB	17.6 C	5.0 D	3.88 B	0.63 C	6	3.25 A	2
5A	SRS log 6.0	1.75 B	22.2 B	5.7 CD	2.88 C	1.75 B	2	2.25 B	3
5B	SRS log 8.0	2.00 AB	19.5 BC	5.1 D	3.13 BC	1.25 BC	2	2.50 B	2
	LSD 0.01	0.71	2.7	1.0	0.76	0.80	NA	0.89	NA

1 Strains of fluorescent pseudomonads used in each treatment are shown in Table 1.

2 HRZ = Inside rhizomes of healthy-appearing ferns from a fernery in Florida without history of Benlate use; SRS = Rhizosphere (roots and rhizomes) of symptomatic ferns in Costa Rica; SRZ = inside rhizomes of symptomatic ferns in Costa Rica.

3 Using the 0 to 3 rating scale described by Kloepper et al. [1].

4 Mean of 8 replicate plants per treatment. Means followed by different letters are significantly different at *P* = 0.01.

**Figure 3:**
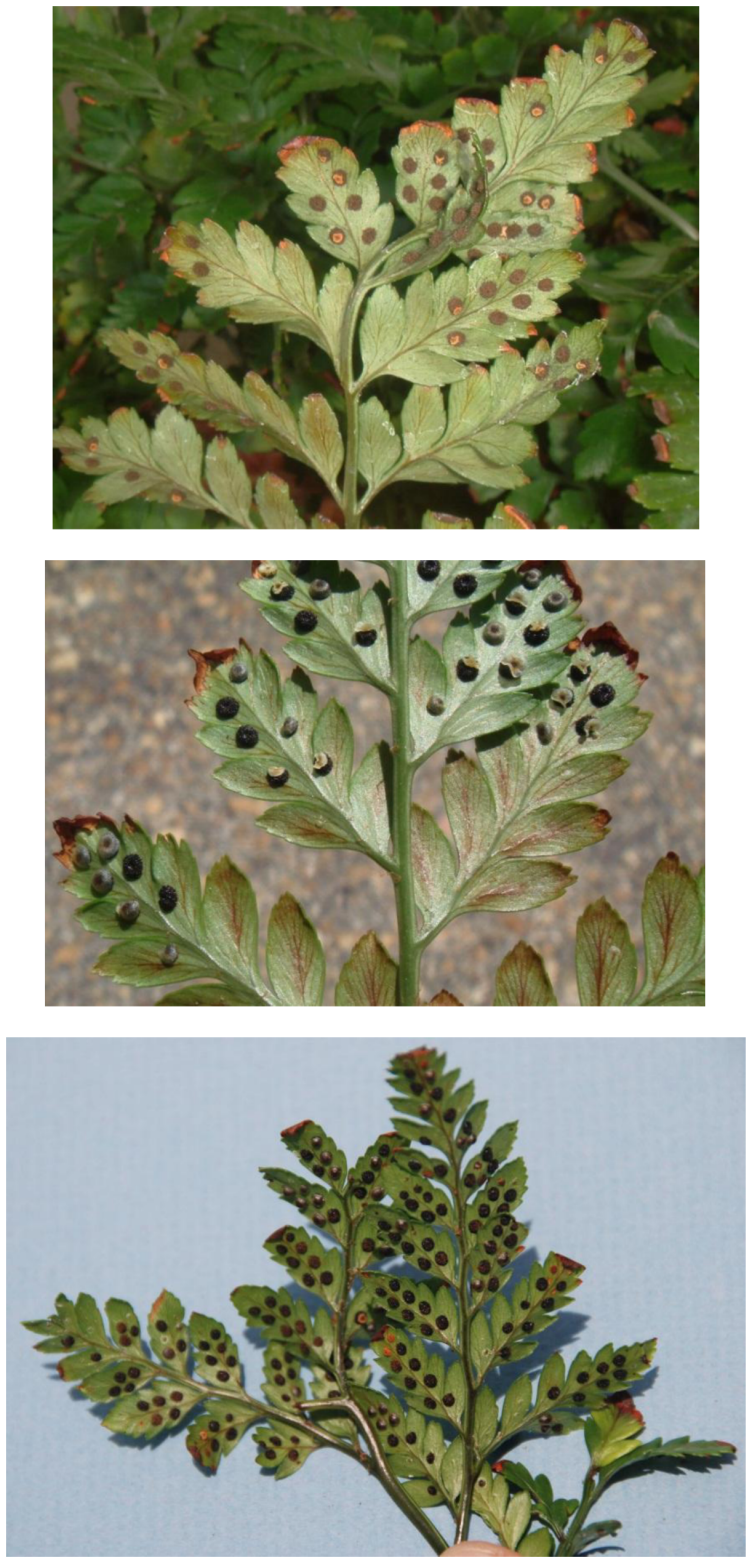
Recreation of FDS symptoms of irregular sporulation. Inoculation with fluorescent pseudomonads from rhizomes of diseased plants. Each image is of a different plant showing asymmetrical pattern of sporulation on lower side of fronds.

**Figure 4:**
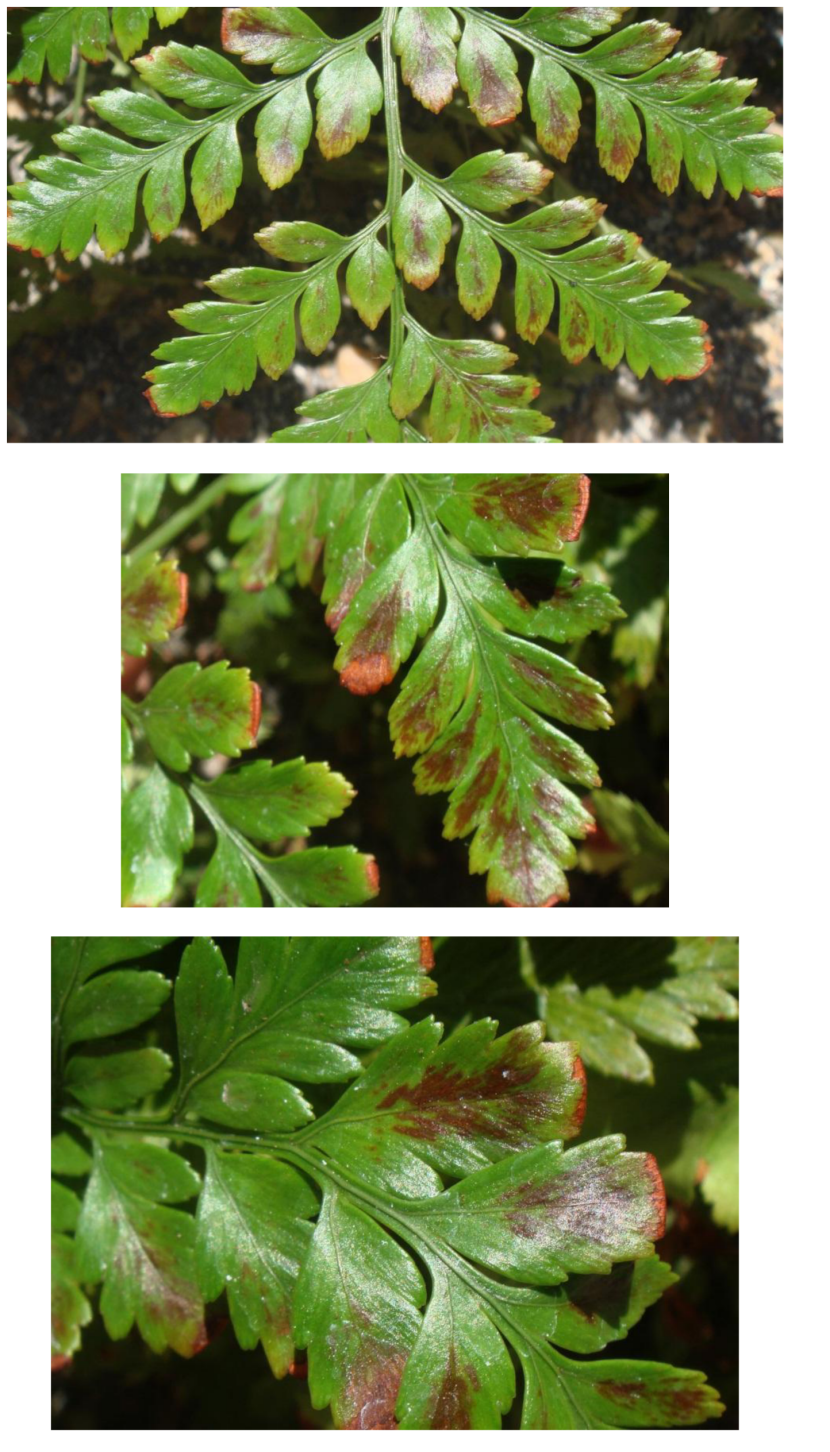
Recreation of FDS symptoms of vena roja. Inoculation with fluorescent pseudomonads from rhizomes of diseased plants. Each image is of a different plant showing reddish streaking of pinnae.

**Figure 5:**
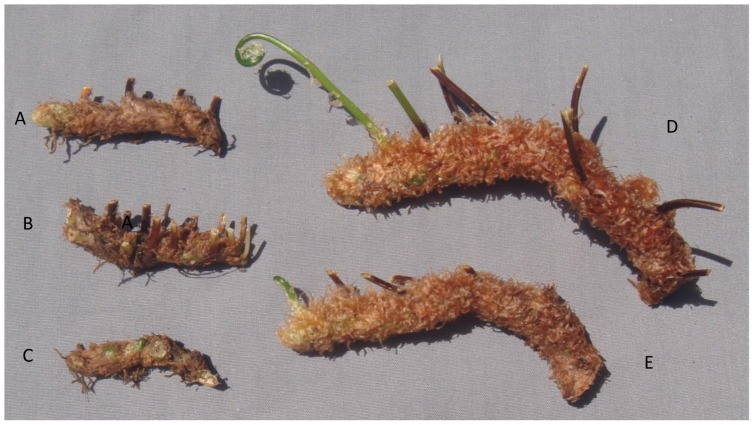
Recreation of FDS symptoms of reduced rhizome diameter. Inoculation with fluorescent pseudomonads from rhizomes and the rhizosphere of diseased plants. Representative examples of rhizomes on ferns 17 months after inoculation. A, B = bacteria from inside rhizomes of ferns with FDS symptoms (treatments 3A and 4A), C = rhizosphere bacteria from ferns with FDS symptoms (treatment 5A), D = bacteria from inside rhizomes of healthy ferns (treatment 2B), E = water control.

**Figure 6:**
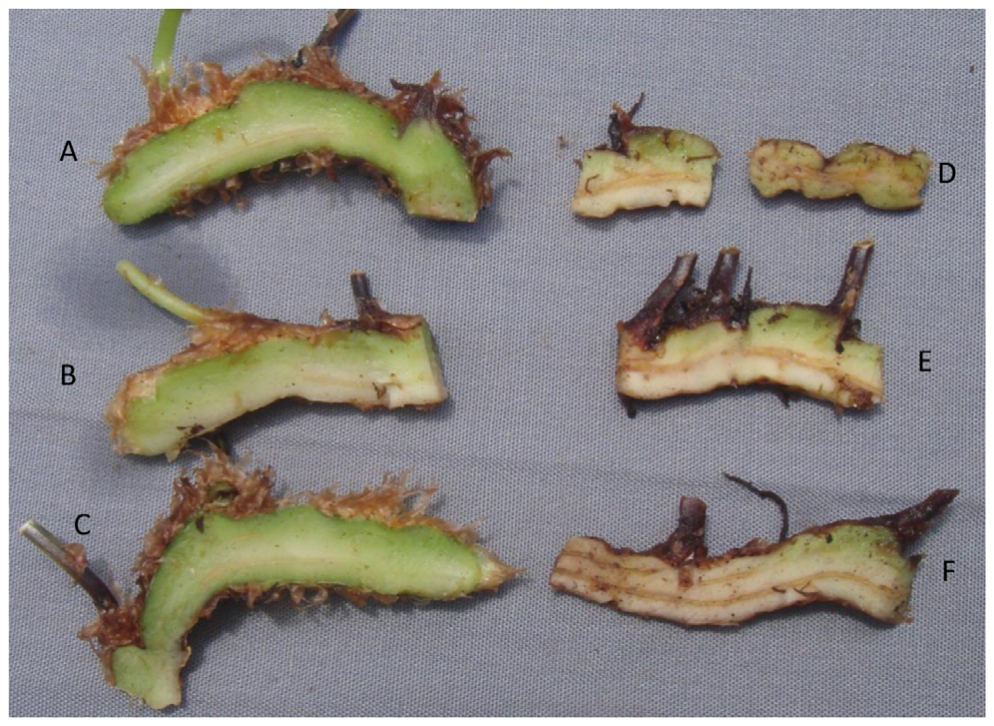
Recreation of FDS symptoms of internal discoloration of rhizomes. Inoculation with fluorescent pseudomonads from rhizomes and the rhizosphere of diseased plants. A, B = bacteria from inside rhizomes of healthy ferns (treatments 2A and 2B), C = water control, D, E = bacteria from inside rhizomes of ferns with FDS symptoms (treatments 3A and 4A), F = rhizosphere bacteria from ferns with FDS symptoms (treatment 5A).

In addition to the main symptom of distorted frond growth, other symptoms associated with FDS include thickening of the fronds resulting in a crispy texture, reduced overall growth ranging to dwarfing of plants, presence of red or yellow streaking of the pinnae, irregular sporulation pattern on the underside of fronds, reduced rhizome diameter, and internal discoloration of rhizomes giving rise to distorted fronds [1]. Each of these symptoms was observed at 12 or 17 months after inoculation with strains of fluorescent pseudomonads from ferns with FDS symptoms but not from the controls (both water-treated and inoculated with bacteria from healthy ferns) (Table 3, Figures 3, 4, 5, and 6). Specifically, thickening of fronds and a crispy texture occurred on 25–37.5% of plants inoculated with bacteria from diseased ferns but on none of the controls; dwarfing developed for 25–75% of plants inoculated with bacteria from diseased ferns but for none of the controls; and significantly reduced growth, indicated both by the mean height of plants and the width of the 5 largest fronds, resulted from all treatments inoculated with bacteria from diseased ferns compared to both the water control and to inoculation with bacteria from healthy ferns (Table 3). Some fronds in treatments inoculated with fluorescent pseudomonads from inside rhizomes of diseased ferns developed irregular sporulation (Figure 3) or reddish streaking of pinnae (Figure 4), a symptom referred to as vena roja in Costa Rica [1]. The below-ground symptoms of reduced rhizome diameter (Figure 5) and internal discoloration of rhizomes (Figure 6) occurred 17 months after inoculation with pseudomonads isolated from inside rhizomes and from the rhizosphere of diseased ferns but not with the water control or with pseudomonads isolated from inside healthy rhizomes.

Data collected during the final destructive sampling 17 months after inoculation (Table 4) further demonstrate that inoculation with fluorescent pseudomonads from diseased ferns led to a significant decline in overall plant growth and vigor. For example, the number of rhizomes per plant, caliper of rhizomes, total plant fresh weight, and dry weights of roots and shoots were significantly (*P* = 0.01) reduced by inoculation with all six treatments of pseudomonads from diseased plants compared to inoculation with the two treatments of pseudomonads from healthy plants and the water control (Table 4).

**Table 4:** Plant growth parameters and internal rhizome populations 17 months after inoculation with fluorescent pseudomonads.

Treatment number^1^	Source of bacteria and concentration 2	Total plant fresh wt. (g)^3^	No. of rhizomes per plant^3^	Caliper of three rhizomes per plant (mm)^3^	Root dry wt. (g)^3^	Shoot dry wt. (g)^3^	Endophytic population of fluorescent pseudomonads inside rhizomes (Log cfu/g )^3^
1	No bacteria	936 a	11.5 ab	7.28 ab	122.8 a	52.8 a	1.36 c
2A	HRZ log 6.0	1101 a	13.4 a	7.90 a	146.8 a	54.6 a	1.34 c
2B	HRZ log 8.0	880 a	9.9 b	6.95 b	120.4 a	48.4 a	1.77 c
3A	SRZ log 6.0	368 b	5.4 c	5.73 c	50.0 b	14.2 b	2.87 b
3B	SRZ log 8.0	280 b	5.0 c	5.51 c	29.2 b	11.6 b	2.95 b
4A	SRZ log 6.0	371 b	5.4 c	5.69 c	40.6b	15.2 b	3.26 ab
4B	SRZ log 8.0	275 b	5.5 c	5.33 c	35.2 b	11.6 b	3.91 ab
5A	SRS log 6.0	353 b	5.0 c	5.47 c	38.6 b	16,0 b	3.89 ab
5B	SRS log 8.0	290 b	5.6 c	5.15 c	28.8 b	10.8 b	4.10 a
	LSD_0.01_	252	1.9	0.86	27.4	11.8	1.09

1 Strains of fluorescent pseudomonads used in each treatment are shown in Table 1.

2 HRZ = Inside rhizomes of healthy-appearing ferns from a fernery in Florida without history of Benlate use; SRS = Rhizosphere (roots and rhizomes) of symptomatic ferns in Costa Rica; SRZ = inside rhizomes of symptomatic ferns in Costa Rica.

3 Mean of 8 replicate plants per treatment. Means followed by different letters are significantly different at *P* = 0.01.

### Reisolation of fluorescent pseudomonads 17 months after inoculation of healthy rhizomes: quantification of endophytic populations inside rhizomes, identification, and characterization

Quantification of fluorescent pseudomonads inside rhizomes of all nine treatments was determined 17 months after inoculation (Table 4). It is important to note that these isolations were performed on new rhizomes that developed on the fern plants that grew from each of the inoculated rhizomes. Populations of fluorescent pseudomonads in the water control and inside rhizomes from plants inoculated with pseudomonads from healthy ferns were significantly lower than populations from all treatments inoculated with pseudomonads from diseased ferns (Table 4). The magnitude of increases in population density compared to the water control ranged from log 1.51 for treatment 3A to log 2.74 for treatment 5B, which correspond to increases of 32-fold and 550-fold, respectively. It is important to note that the final populations of fluorescent pseudomonads in plants inoculated with strains from inside rhizomes of healthy ferns (treatments 2A and 2B in Table 4) were statistically equivalent to those inside the non-inoculated, water control. This finding supports the conclusion that the capacity of FPs to enter, colonize, and persist in rhizomes is strain-specific.

**Figure 7:**
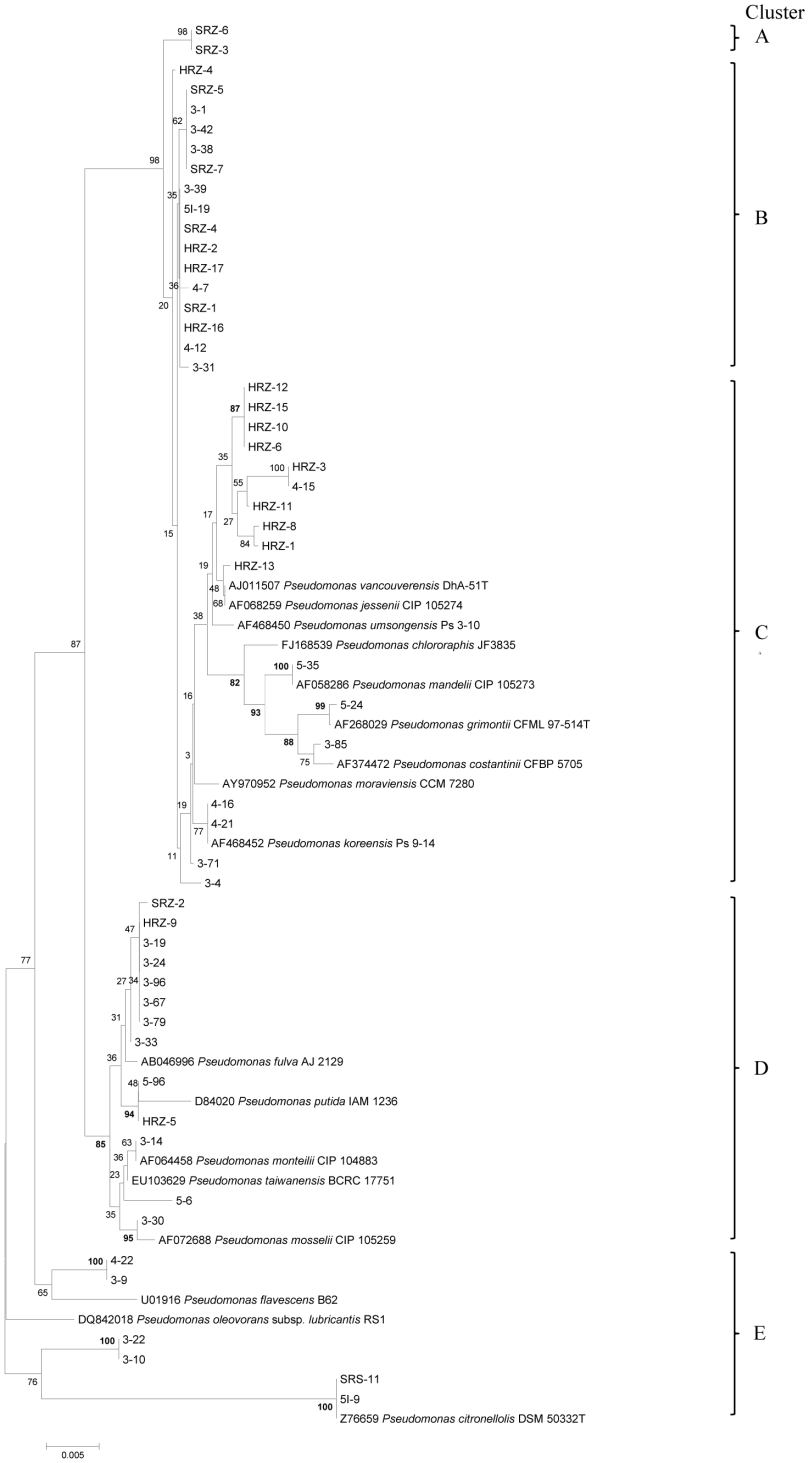
Neighbor-joining (NJ) bootstrap consensus tree of partial 16S rRNA gene (1412 bp) illustrating phylogenetic relationship among fluorescent *Pseudomonas* spp. isolated from inside rhizomes 17 months after inoculation. The first number in strain codes indicates the treatment number from the inoculation experiment (Table 2). The analyses were done with MEGA version 5 [6], and the numbers at each node represent bootstrap value (1000 replicates). Scale bar: substitutions/site.

To determine if the same bacteria inoculated into rhizomes could be recovered from symptomatic plants, 350 strains were isolated from inside newly formed rhizomes of treatments 3, 4, and 5 at 17 months after inoculation, as described in the methods. The phylogenetic analyses of these reisolated strains were compared to the corresponding analyses of the strains that were used to inoculate rhizomes to recreate symptoms of FDS. Overall, the results (Figure 7) indicate that some of the inoculated strains were recovered from ferns that developed symptoms. Specifically, the reisolated strains fit into four phylogenetic clusters. Three of these clusters (B, D, and E) were the same as the inoculated strains originally isolated from symptomatic ferns in Costa Rica (Table 5). The predominant phylogenetic cluster was B, which accounted for more than 50% of the 350 reisolated strains from among all four treatments.

**Table 5:** Summary of the percentage of fluorescent pseudomonads reisolated in each treatment group belonging to phylogenetic clusters of the inoculated strains shown in Figure 1.

	Percentage of reisolated strains from each treatment belonging to the phylogenetic clusters of the inoculated strains				
	Treatment				
Phylogenetic Clusters	SRZ (trt 3)	SRZ (trt 4)	SRS (trt 5)	SRS-INT^1^ (trt 5)	Closest matches of type strains with highest SeqMatch score^2^
**A**	0	0	0	0	
**B**	26	76	47	72	*Pseudomonas moraviensis* (0.974); *Pseudomonas koreensis* (0.964); *Pseudomonas umsongensis* (0.953); *Pseudomonas vancouverensis* (0.951)
**C**	12	22	26	0	*Pseudomonas koreensis* (1.000); *Pseudomonas vancouverensis* (0.977); *Pseudomonas umsongensis* (0.972); *Pseudomonas jessenii* (0.966); *Pseudomonas mandelii* (0.997); *Pseudomonas grimontii* (0.995); *Pseudomonas costantinii* (0.974)
**D**	40	1	24	0	*Pseudomonas taiwanensis* (0.996); *Pseudomonas monteilii* (0.990); *Pseudomonas mosselii* (0.989); *Pseudomonas fulva/putida* (0.965)
**E**	21	1	3	26	*Pseudomonas citronellolis* (0.981); *Pseudomonas flavescens* (0.912); *Pseudomonas oleovorans* (0.912)

1 INT indicates isolates obtained following printing of rhizomes showing internal discoloration as described in the methods.

2 Taxa of fluorescent pseudomonads in the Ribosomal Database Project II with 16S rRNA gene sequences most similar to those isolates in each cluster.

It is also interesting to compare the percentages of reisolated strains from treatments 5 and 5INT. Both of these treatments were inoculated with the same group of fluorescent pseudomonads from the rhizosphere of symptomatic ferns, but 5INT was reisolated by printing cross sections of rhizomes showing internal necrosis onto agar as described in the methods. From 5-INT, only two phylogenetic clusters were found (B and E) (Table 5). In addition, 13 strains reisolated from 5-INT were identical to the two strains from cluster E that were in the inoculum (Table 2). Collectively, these results suggest that the specific symptom of internal necrosis might be caused by members of clusters B and E, a proposition which should be further investigated.

In addition to analyzing the 350 reisolated strains phylogenetically, we also compared their physiological profiles in comparison to the inoculated strains by evaluating production of HR, pectinolytic enzymes, and IAA. The results indicated that HR was elicited in 24–48 hours by 77% of the inoculated strains (treatments 3+4+5 in Table 1) and by 50% of the strains reisolated from inside rhizomes (treatments 3+4+5 in Table S1). Interestingly, the frequency of elicitation of HR was higher (62%) for treatment 5INT (Table S1), which included strains isolated by pressing onto agar cross sections of rhizomes showing internal discoloration (Figure 6D). Similarly, strong pectinolytic activity, indicated by rot of potato slice within 24 hours, was exhibited by 17% of the inoculated strains (treatments 3+4+5 in Table 1) and by 34% of the strains reisolated from inside rhizomes (treatments 3+4+5 in Table S1). As occurred with frequency of HR, the frequency of strong pectinolytic activity was higher (66%) for treatment 5INT (Table S1). IAA was produced at average levels of 8.4 to12.0 µg/ml of strains from the three inoculated treatments (treatments 3, 4, and 5 in Table 1) and by 8.5 to12.3 µg/ml of the reisolated strains.

## Discussion

The results from the identification of fluorescent pseudomonads isolated from field-grown ferns in Costa Rica show that healthy and diseased ferns have different communities of pseudomonads inside rhizomes and in the rhizosphere. For example, identification of the strains based on 16S rRNA gene analysis (Table 1, Figure 1, and Table 2) demonstrated a trend to reduced bacterial diversity inside rhizomes of diseased ferns compared to inside healthy fern rhizomes. Hence, during development of FDS, some phylogenetic clusters became dominant inside fern rhizomes, while strains belonging to the dominant phylogenetic cluster of pseudomonads inside healthy rhizomes were not detected inside rhizomes from diseased ferns.

Characterization of the pseudomonad strains (Table 1) indicated that in addition to differences in the phylogenetic groups, fluorescent pseudomonads from diseased and healthy ferns differed functionally. Strains from diseased ferns had higher frequencies of elicitation of the hypersensitive response in tobacco, which is an indication that a bacterial strain is pathogenic [6]. The finding that 46% of the strains isolated from inside rhizomes or from the rhizosphere of diseased ferns exhibited strong pectinolytic activity by causing soft rot of potato slices in 24 hours compared to none of the strains from healthy ferns was surprising. Pectinolytic enzymes are a virulence factor for some pathogens such as soft-rotting bacteria. Interestingly, one of the secondary symptoms sometimes associated with FDS is an internal discoloration of rhizomes (Figure 6) that resembles soft rot of potato tubers. In addition to being a virulence factor for some pathogens, pectinolytic enzymes can facilitate entry of rhizosphere bacteria inside plants by hydrolyzing pectic substances in the middle lamella of the plant outer cell wall [12]. Pectinolytic enzymes have also been suggested to be a possible mechanism by which deleterious rhizobacteria cause plant damage [13].

In contrast to elicitation of HR and pectinolytic enzyme activity, the level of IAA production was not less for strains isolated from healthy than from diseased plants. Production of IAA has been reported to be a determinant of allelopathic or deleterious effects of some rhizosphere bacteria on plants [14] and [3]. Lindow et al. [15] reported that fruit russet of pear was associated with mixed populations of IAA-producing epiphytic bacteria, including species of fluorescent pseudomonads. It is possible that IAA production by endophytic pseudomonads contributes to the symptoms of FDS because bacterial production of IAA is regulated by quorum sensing [16]. Hence, as endophytic populations of pseudomonads inside rhizomes increase, as occurred following inoculation in our study, IAA production *in planta* would be expected to increase.

The strains of fluorescent pseudomonads isolated from the rhizosphere and inside rhizomes of diseased ferns but not those isolated from healthy ferns recreated the main symptoms of FDS 12 months after inoculation in the greenhouse study (Figure 2). Leatherleaf fern plants grow slowly when first started from an individual rhizome, as in our study. The first symptoms of distorted fronds were noted about five months after inoculation, when the third new fronds were developing on the newly planted rhizomes, and we waited an additional 7 months to rate symptoms in order to simulate a growing season in the field. These main symptoms included twisting and deformations of fronds and loss of the normal pyramidal frond shape for which Leatherleaf fern is highly valued. It was surprising to see the extent of secondary symptoms of FDS which were also reproduced following inoculation of rhizomes with bacteria from diseased ferns. For example, thickening of fronds resulting in a crispy texture, reduced overall growth sometimes resulting in dwarfing of plants, the presence of red or yellow streaks on the pinnae of fronds, an irregular pattern of sporulation, reduced size of new rhizomes, and internal discoloration of rhizomes were noted in some plants inoculated with strains of pseudomonads isolated from diseased plants, while ferns inoculated with fluorescent pseudomonads from healthy ferns did not have these symptoms. This production of the main and secondary symptoms of FDS following inoculation lends support to the idea that the secondary symptoms reported by growers in Costa Rica [1] are actually part of the same disease syndrome, FDS, as the main symptoms of frond distortions.

As described in the results, a collection of pseudomonads was obtained directly from cross sections of new rhizomes that exhibited internal necrosis 17 months after inoculation. The phylogenetic analysis of these strains revealed that only two of the inoculated clusters were represented by the strains inside the zone of necrosis. One interpretation of this finding could be that some of the secondary symptoms are caused by specific strains within the community of harmful endophytic bacteria associated with FDS. Further studies aimed at elucidating how mixed genotypes of fluorescent pseudomonads produce diverse symptoms are needed to understand fully how the presence of secondary symptoms varies based on genotypes of endophytic pseudomonads present and climate such as altitude and annual rainfall, two climatic conditions that vary across Leatherleaf fern production areas.

It is important to note that the genus *Pseudomonas* contains a large diversity of species and strains within species [17] and that within the fluorescent pseudomonads are many biological control strains as well as deleterious and pathogenic strains. Loper et al. [18] sequenced the genome of seven beneficial plant-associated strains in the *P. fluorescens* group and found that only about half of the genome was common among all the strains. Interestingly, in the study of Loper el al. [18], none of the beneficial strains of *Pseudomonas* elicited HR and only one demonstrated pectinolytic activity on potato slice, results which are consistent with our finding that elicitation of HR and pectinolytic activity was common among strains from diseased but not healthy ferns. It is also important to note that while the emphasis of this study was on the role of fluorescent pseudomonads in the etiology of FDS, it is possible that other culturable and nonculturable bacteria also contribute to production of some of the diverse symptoms of FDS.

## Conclusions

Overall, the results reported here clearly demonstrate that the bacteria isolated from diseased fern plants in Costa Rica recreated the main reported symptoms [1] of FDS, while the water control and the bacteria isolated from healthy plants did not. Interestingly, inoculation with bacteria from healthy plants actually significantly (*P* = 0.01) increased one parameter of plant growth, frond width, compared to the water control (Table 3) (Figure 2B). This finding shows that some endophytic bacteria inside rhizomes of healthy plants are beneficial to plant development, which is consistent with studies on PGPR (plant growth-promoting rhizobacteria).

As pointed out by Preston [17], the distinction between saprophytes and pathogens is not always clear-cut because both live on and inside plant tissues where there are frequent opportunities for recombination after horizontal gene transfer, a process which confers new phenotypic traits (or suites of traits) to bacteria sharing an ecological habitat [19]. Hence, genes for production of virulence factors may move among species of phylogenetic groups of bacteria in the rhizosphere or inside plants, and expression of the virulence factors can relate to the population density of the bacterial strains. For example, several virulence factors have been shown to be regulated by bacterial cell density via quorum sensing, including elicitation of HR [16], production of IAA [17], and production of cell wall degrading enzymes including pectinase [19], [20]. In addition, quorum sensing regulates both horizontal gene transfer and bacterial colonization of plant hosts [19]. Based upon these reports and our results, we propose the following model for how fluorescent pseudomonads cause FDS. Among the native population of bacteria in the rhizosphere and inside rhizomes of healthy ferns are groups of fluorescent pseudomonads, and likely other eubacterial genera, that contain genes for production of virulence and colonization factors. Perturbations in the growth of ferns, such as the application of specific systemic fungicides [3], trigger population increases of these bacterial groups. As populations increase, quorum sensing activates expression of colonization and virulence factors as well as horizontal transfer of virulence genes to other closely related phylogenetic groups. Over time, there is a shift in bacterial community inside rhizomes with a concomitant development of symptoms of FDS. This model is consistent with previous studies reviewed in Berg et al. [19] that the rhizosphere is a reservoir of opportunistic pathogens that cause human infections.

## Supporting Information

Table S1 Details of identification and characterization of 350 fluorescent pseudomonads isolated from inside rhizomes of fern expressing symptoms of FDS 17 months after inoculation.
(DOCX)
