# Supplementary material for: Symptoms of Fern Distortion Syndrome Resulting from Inoculation with Opportunistic Endophytic Fluorescent Pseudomonas spp
Source: PLoS One. 2013 Mar 13;8(3):e58531. doi: 10.1371/journal.pone.0058531 (PMC3596302; doi:10.1371/journal.pone.0058531)
Supplement: Table S1 — Details of identification and characterization of 350 fluorescent pseudomonads isolated from inside rhizomes of fern expressing symptoms of FDS 17 months after inoculation. (DOCX) [file pone.0058531.s001.docx]

**Table S1.** Details of identification and characterization of 350 fluorescent pseudomonads isolated from inside rhizomes of fern expressing symptoms of FDS 17 months after inoculation.

| Treatment^1^ and strain number | Phylogenetic Cluster^2^ | HR in tobacco^3^ | Rot of potato slice^4^ | IAA production |
| --- | --- | --- | --- | --- |
| **3-1** | B | + | ++ | 9.06 |
| 3-2 | D | ++ | + | 16.42 |
| 3-3 | B | ++ | ++ | 6.60 |
| **3-4** | C | + | - | 7.24 |
| 3-5 | D | + | - | 18.47 |
| 3-6 | D | ++ | - | 17.87 |
| 3-7 | B | ++ | ++ | 6.50 |
| 3-8 | C | ++ | - | 7.15 |
| **3-9** | E | - | - | 19.89 |
| **3-10** | E | + | - | 1.44 |
| 3-11 | E | - | - | 0.89 |
| 3-12 | C | - | + | 6.71 |
| 3-13 | C | + | - | 6.41 |
| **3-14** | D | + | - | 16.68 |
| 3-15 | D | + | + | 17.74 |
| 3-16 | E | - | - | 1.30 |
| 3-17 | D | - | - | 14.60 |
| 3-18 | D | - | - | 18.94 |
| **3-19** | D | - | - | 24.54 |
| 3-20 | D | ++ | + | 16.63 |
| 3-21 | C | - | - | 19.47 |
| **3-22** | E | ++ | - | 1.51 |
| 3-23 | E | - | - | 1.45 |
| **3-24** | D | + | - | 18.44 |
| 3-25 | B | - | + | 8.18 |
| 3-26 | E | - | - | 0.78 |
| 3-27 | D | - | - | 16.19 |
| 3-28 | D | + | - | 17.07 |
| 3-29 | D | + | - | 19.05 |
| **3-30** | D | ++ | ++ | 14.08 |
| **3-31** | B | - | - | 7.91 |
| 3-32 | B | - | - | 8.28 |
| **3-33** | D | - | - | 15.73 |
| 3-35 | E | - | - | 0.61 |
| 3-36 | D | - | - | 14.85 |
| 3-37 | B | - | - | 11.47 |
| **3-38** | B | - | ++ | 8.28 |
| **3-39** | B | + | ++ | 7.84 |
| 3-40 | B | - | ++ | 7.52 |
| 3-41 | B | - | ++ | 7.02 |
| **3-42** | B | - | ++ | 7.55 |
| 3-43 | D | - | - | 25.63 |
| 3-44 | D | - | - | 28.11 |
| 3-45 | B | - | ++ | 8.60 |
| 3-46 | B | - | ++ | 9.32 |
| 3-47 | B | + | ++ | 8.45 |
| 3-48 | B | - | ++ | 10.12 |
| 3-49 | C | ++ | ++ | 7.86 |
| 3-50 | D | ++ | - | 26.34 |
| 3-51 | B | + | - | 10.90 |
| 3-52 | E | - | + | 0.75 |
| 3-53 | E | + | - | 0.94 |
| 3-54 | E | + | - | 1.07 |
| 3-55 | E | - | - | 0.87 |
| 3-56 | D | ++ | - | 8.47 |
| 3-57 | E | - | - | 0.94 |
| 3-58 | E | ++ | - | 0.78 |
| 3-59 | E | + | - | 0.67 |
| 3-60 | E | ++ | - | 1.86 |
| 3-61 | D | + | - | 21.48 |
| 3-62 | E | - | - | 1.47 |
| 3-63 | E | - | - | 1.49 |
| 3-64 | D | + | + | 12.33 |
| 3-65 | D | - | - | 11.99 |
| 3-66 | D | - | - | 12.55 |
| **3-67** | D | + | - | 16.88 |
| 3-68 | D | + | + | 18.31 |
| 3-69 | D | ++ | - | 12.54 |
| 3-70 | D | + | - | 16.95 |
| **3-71** | C | + | - | 20.24 |
| 3-72 | E | - | - | 0.98 |
| 3-73 | E | + | - | 1.14 |
| 3-74 | D | + | - | 13.75 |
| 3-75 | B | - | + | 8.95 |
| 3-76 | D | - | - | 18.16 |
| 3-77 | D | - | - | 19.81 |
| 3-78 | D | - | - | 20.37 |
| **3-79** | D | - | + | 22.17 |
| 3-80 | C | - | - | 8.85 |
| 3-81 | D | + | - | 25.61 |
| 3-82 | D | + | + | 16.68 |
| 3-83 | C | ++ | + | 6.09 |
| 3-84 | D | ++ | - | 25.27 |
| **3-85** | C | ++ | - | 15.77 |
| 3-86 | B | + | ++ | 9.49 |
| 3-87 | B | - | ++ | 10.85 |
| 3-88 | B | - | ++ | 10.12 |
| 3-89 | B | ++ | ++ | 6.78 |
| 3-90 | B | + | ++ | 7.19 |
| 3-91 | E | + | - | 1.40 |
| 3-92 | C | + | - | 6.24 |
| 3-93 | D | ++ | + | 19.58 |
| 3-94 | C | ++ | - | 6.81 |
| 3-95 | D | ++ | + | 19.01 |
| **3-96** | D | ++ | - | 13.40 |
| 3-97 | D | ++ | - | 17.94 |
| 3-98 | B | ++ | ++ | 5.30 |
| 3-99 | B | ++ | ++ | 6.03 |
| 3-100 | B | - | + | 5.23 |
| 4-1 | B | + | ++ | 5.01 |
| 4-2 | B | + | ++ | 5.12 |
| 4-3 | B | - | ++ | 6.10 |
| 4-4 | B | + | ++ | 14.43 |
| 4-5 | B | - | - | 11.93 |
| 4-6 | B | - | ++ | 9.79 |
| **4-7** | B | - | - | 12.83 |
| 4-8 | B | - | ++ | 6.76 |
| 4-9 | B | - | ++ | 7.27 |
| 4-10 | B | + | + | 8.39 |
| 4-11 | B | - | ++ | 9.80 |
| **4-12** | B | - | ++ | 14.48 |
| 4-13 | D | - | - | 22.64 |
| 4-14 | B | - | ++ | 16.93 |
| **4-15** | C | + | - | 33.64 |
| **4-16** | C | - | - | 11.95 |
| 4-17 | C | - | - | 11.63 |
| 4-18 | C | ++ | + | 11.91 |
| 4-19 | B | ++ | + | 12.83 |
| 4-20 | C | + | + | 8.00 |
| **4-21** | C | - | - | 13.11 |
| **4-22** | E | - | - | 29.87 |
| 4-23 | B | - | + | 13.50 |
| 4-24 | B | + | + | 17.09 |
| 4-25 | B | - | + | 18.13 |
| 4-26 | B | - | ++ | 16.46 |
| 4-27 | B | + | ++ | 18.54 |
| 4-28 | B | + | ++ | 6.62 |
| 4-29 | B | - | ++ | 6.46 |
| 4-30 | B | ++ | ++ | 6.09 |
| 4-31 | B | - | + | 6.25 |
| 4-32 | B | - | ++ | 5.90 |
| 4-33 | B | - | ++ | 6.45 |
| 4-34 | C | - | + | 6.55 |
| 4-35 | B | - | + | 10.80 |
| 4-36 | B | - | ++ | 7.05 |
| 4-37 | B | ++ | ++ | 6.17 |
| 4-38 | C | - | - | 6.10 |
| 4-39 | B | - | + | 8.35 |
| 4-40 | B | - | - | 6.03 |
| 4-41 | C | - | - | 6.78 |
| 4-42 | B | - | - | 5.35 |
| 4-43 | B | - | ++ | 5.04 |
| 4-44 | B | ++ | ++ | 5.11 |
| 4-45 | C | - | - | 5.51 |
| 4-46 | B | - | - | 5.44 |
| 4-47 | B | - | ++ | 5.51 |
| 4-48 | B | - | - | 5.90 |
| 4-49 | B | - | ++ | 5.37 |
| 4-50 | B | - | ++ | 5.88 |
| 4-51 | B | ++ | + | 5.96 |
| 4-52 | B | - | - | 8.85 |
| 4-53 | C | + | - | 6.31 |
| 4-54 | C | - | - | 5.81 |
| 4-55 | B | - | - | 5.97 |
| 4-56 | B | - | + | 5.53 |
| 4-57 | B | - | - | 5.58 |
| 4-58 | C | - | + | 6.30 |
| 4-59 | B | - | ++ | 6.00 |
| 4-60 | C | + | - | 11.89 |
| 4-61 | C | - | - | 6.30 |
| 4-62 | B | - | - | 5.93 |
| 4-63 | B | - | - | 6.57 |
| 4-64 | B | + | ++ | 5.51 |
| 4-65 | C | - | - | 6.39 |
| 4-66 | B | + | - | 9.63 |
| 4-67 | B | - | ++ | 6.34 |
| 4-68 | B | - | ++ | 10.55 |
| 4-69 | B | + | ++ | 6.03 |
| 4-70 | B | - | ++ | 6.44 |
| 4-71 | C | - | + | 7.57 |
| 4-72 | B | + | - | 5.92 |
| 4-73 | B | + | ++ | 11.38 |
| 4-74 | B | + | ++ | 7.57 |
| 4-75 | C | - | - | 6.41 |
| 4-76 | B | ++ | ++ | 5.63 |
| 4-77 | B | ++ | ++ | 5.93 |
| 4-78 | B | + | ++ | 6.13 |
| 4-79 | B | - | + | 8.45 |
| 4-80 | B | ++ | ++ | 6.14 |
| 4-81 | B | - | ++ | 6.53 |
| 4-82 | B | + | ++ | 5.86 |
| 4-83 | C | + | - | 6.60 |
| 4-84 | B | - | + | 7.68 |
| 4-85 | B | - | ++ | 5.68 |
| 4-86 | C | - | - | 6.21 |
| 4-87 | C | - | ++ | 5.83 |
| 4-88 | B | + | - | 5.56 |
| 4-89 | B | ++ | ++ | 6.03 |
| 4-90 | B | - | - | 5.68 |
| 4-91 | B | + | ++ | 5.90 |
| 4-92 | B | + | ++ | 5.81 |
| 4-93 | B | - | + | 5.65 |
| 4-94 | B | - | + | 8.43 |
| 4-95 | C | + | - | 6.09 |
| 4-96 | B | - | ++ | 5.79 |
| 4-97 | B | - | + | 6.81 |
| 4-98 | B | - | + | 6.23 |
| 4-99 | B | - | ++ | 7.96 |
| 4-100 | B | + | + | 11.36 |
| 5-1 | B | - | - | 9.12 |
| 5-2 | B | + | ++ | 11.13 |
| 5-3 | B | + | + | 10.65 |
| 5-4 | B | - | + | 7.24 |
| 5-5 | B | - | ++ | 7.38 |
| **5-6** | D | ++ | - | 8.65 |
| 5-7 | B | + | ++ | 7.66 |
| 5-8 | B | + | + | 7.26 |
| 5-9 | B | - | ++ | 7.37 |
| 5-10 | B | - | ++ | 7.71 |
| 5-11 | B | - | - | 11.84 |
| 5-12 | B | - | - | 11.85 |
| 5-13 | B | - | - | 11.39 |
| 5-14 | D | - | - | 9.81 |
| 5-15 | D | - | - | 11.31 |
| 5-16 | B | - | ++ | 8.19 |
| 5-17 | B | - | ++ | 8.36 |
| 5-18 | B | - | ++ | 8.17 |
| 5-19 | B | - | + | 7.52 |
| 5-20 | B | - | ++ | 7.97 |
| 5-21 | B | - | ++ | 7.64 |
| 5-22 | B | - | ++ | 8.29 |
| 5-23 | B | - | ++ | 7.55 |
| **5-24** | C | - | ++ | 6.94 |
| 5-25 | C | - | ++ | 6.88 |
| 5-26 | B | - | ++ | 7.08 |
| 5-27 | C | - | ++ | 6.74 |
| 5-28 | C | - | + | 6.94 |
| 5-29 | E | - | - | 1.84 |
| 5-30 | B | - | ++ | 9.59 |
| 5-31 | C | + | + | 7.50 |
| 5-32 | B | ++ | ++ | 9.74 |
| 5-33 | C | ++ | - | 7.59 |
| 5-34 | B | ++ | ++ | 9.48 |
| **5-35** | C | ++ | - | 13.34 |
| 5-36 | C | ++ | + | 13.89 |
| 5-37 | C | ++ | - | 12.20 |
| 5-38 | C | ++ | + | 11.64 |
| 5-39 | C | ++ | + | 6.91 |
| 5-40 | C | ++ | - | 14.00 |
| 5-41 | C | + | ++ | 10.72 |
| 5-42 | B | - | - | 7.58 |
| 5-43 | C | + | + | 7.82 |
| 5-44 | C | - | + | 9.05 |
| 5-45 | B | ++ | - | 11.31 |
| 5-46 | B | - | - | 10.37 |
| 5-47 | C | - | ++ | 7.36 |
| 5-48 | C | - | + | 7.78 |
| 5-49 | C | ++ | + | 8.67 |
| 5-50 | C | ++ | - | 7.62 |
| 5-51 | B | ++ | ++ | 8.79 |
| 5-52 | C | ++ | ++ | 7.97 |
| 5-53 | C | - | + | 7.64 |
| 5-54 | B | ++ | ++ | 8.51 |
| 5-55 | E | - | - | 1.63 |
| 5-56 | B | ++ | ++ | 8.07 |
| 5-57 | B | + | - | 8.63 |
| 5-58 | B | + | ++ | 7.93 |
| 5-59 | B | + | ++ | 7.73 |
| 5-60 | B | + | ++ | 7.51 |
| 5-61 | B | + | ++ | 7.69 |
| 5-62 | B | + | ++ | 9.76 |
| 5-63 | B | ++ | ++ | 7.09 |
| 5-64 | C | - | - | 11.89 |
| 5-65 | C | - | - | 19.92 |
| 5-66 | C | - | - | 17.67 |
| 5-67 | C | - | - | 15.86 |
| 5-68 | B | + | ++ | 9.39 |
| 5-69 | B | - | ++ | 10.30 |
| 5-70 | C | - | - | 7.44 |
| 5-71 | B | + | ++ | 10.18 |
| 5-72 | D | - | - | 11.04 |
| 5-73 | B | ++ | ++ | 8.05 |
| 5-74 | B | + | - | 8.08 |
| 5-75 | B | ++ | ++ | 7.26 |
| 5-76 | D | ++ | - | 19.38 |
| 5-77 | D | - | - | 8.64 |
| 5-78 | D | + | + | 19.88 |
| 5-79 | D | + | - | 18.77 |
| 5-80 | D | ++ | - | 10.90 |
| 5-81 | D | - | - | 107.15 |
| 5-82 | B | - | - | 14.13 |
| 5-83 | B | ++ | - | 9.27 |
| 5-84 | D | - | - | 19.39 |
| 5-85 | D | - | - | 18.93 |
| 5-86 | D | + | + | 19.00 |
| 5-87 | E | - | - | 23.93 |
| 5-88 | B | + | ++ | 10.41 |
| 5-89 | B | - | ++ | 11.84 |
| 5-90 | D | - | - | 9.74 |
| 5-91 | D | - | - | 9.24 |
| 5-92 | D | ++ | - | 8.85 |
| 5-93 | D | - | - | 8.96 |
| 5-94 | D | - | - | 17.56 |
| 5-95 | D | - | - | 18.76 |
| **5-96** | D | ++ | + | 18.45 |
| 5-97 | D | ++ | - | 17.98 |
| 5-98 | D | - | + | 18.62 |
| 5-99 | D | - | - | 96.71 |
| 5-100 | D | - | - | 12.05 |
| 5INT-1 | B | - | - | 7.91 |
| 5INT-2 | B | - | ++ | 7.69 |
| 5INT-3 | B | + | ++ | 7.19 |
| 5INT-4 | B | - | ++ | 7.06 |
| 5INT-5 | E | + | - | 7.47 |
| 5INT-6 | B | + | ++ | 7.43 |
| 5INT-7 | B | ++ | ++ | 6.59 |
| 5INT-8 | E | - | + | 8.14 |
| **5INT-9** | E | + | - | 7.69 |
| 5INT-10 | B | ++ | ++ | 7.27 |
| 5INT-11 | B | + | ++ | 7.86 |
| 5INT-12 | B | + | ++ | 7.50 |
| 5INT-13 | B | + | ++ | 8.12 |
| 5INT-14 | E | - | - | 7.33 |
| 5INT-15 | B | + | ++ | 9.10 |
| 5INT-16 | B | ++ | ++ | 11.03 |
| 5INT-17 | E | - | - | 7.84 |
| 5INT-18 | B | - | ++ | 9.69 |
| **5INT-19** | B | + | ++ | 15.72 |
| 5INT-20 | B | + | ++ | 16.49 |
| 5INT-21 | B | - | ++ | 9.69 |
| 5INT-22 | B | + | ++ | 9.74 |
| 5INT-23 | B | - | ++ | 9.45 |
| 5INT-24 | B | - | ++ | 7.78 |
| 5INT-25 | E | + | + | 7.98 |
| 5INT-26 | B | ++ | ++ | 10.59 |
| 5INT-27 | B | ++ | ++ | 8.72 |
| 5INT-28 | B | ++ | ++ | 8.61 |
| 5INT-29 | B | + | ++ | 10.06 |
| 5INT-30 | B | + | ++ | 9.34 |
| 5INT-31 | B | + | ++ | 9.25 |
| 5INT-32 | B | + | ++ | 10.57 |
| 5INT-33 | E | - | - | 10.23 |
| 5INT-34 | E | - | - | 7.68 |
| 5INT-35 | B | + | ++ | 13.44 |
| 5INT-36 | B | ++ | ++ | 11.77 |
| 5INT-37 | B | + | ++ | 7.98 |
| 5INT-38 | B | + | ++ | 7.69 |
| 5INT-39 | E | - | - | 10.71 |
| 5INT-40 | B | + | ++ | 8.17 |
| 5INT-41 | B | ++ | - | 8.32 |
| 5INT-42 | B | ++ | ++ | 8.39 |
| 5INT-43 | B | ++ | + | 9.58 |
| 5INT-45 | B | - | ++ | 9.30 |
| 5INT-46 | B | - | ++ | 7.69 |
| 5INT-47 | E | - | + | 6.83 |
| 5INT-48 | E | + | + | 8.14 |
| 5INT-49 | E | + | ++ | 8.77 |
| 5INT-50 | E | - | - | 8.07 |

^1^ 100 strains were selected from inside rhizomes of treatments 3, 4, and 5. In addition, from treatment 5, 50 isolates (labeled here 5INT) were selected by cutting rhizomes longitudinally, observing vascular discoloration, and pressing the cut surface onto 50% King’s medium B to allow growth and selection of individual colonies of fluorescent pseudomonads. Treatments are listed in Table 1.

^2^ Identified using phylogenetic analysis of 16S rDNA sequencing.

^3^ Tobacco hypersensitive test. ++ indicates dry necrosis in 24-36 hr after inoculation; + indicates wet necrosis in 48 hr.

^4^ Measurement of pectinolytic activity. ++ indicates soft rot of potato slice 24-36 hr after inoculation; + indicates soft rot 48-60 hr after inoculation.
